# Supplementary material for: Chikungunya Virus RNA Secondary Structures Impact Defective Viral Genome Production
Source: Microorganisms. 2024 Aug 29;12(9):1794. doi: 10.3390/microorganisms12091794 (PMC11434300; doi:10.3390/microorganisms12091794)
Supplement: Supplementary file 1 [file microorganisms-12-01794-s001.zip › microorganisms-3130558-supplementary.pdf]

Supplementary Figure S1: D2S mutant and WT virus MOI and median SHAPE across passages

A, High MOI passages (MOI 10 for P1 followed by blind passages with high volume) were performed with WT CHIKV Carib (black line) and D2S mutant (blue line) in Vero cells. Bars represent mean ± SD, *n* = 6 biological replicates; NS, not significant (two-way ANOVA with Sidak's multiple comparison).

B, Across all passages, nucleotides that were used as start and/or stop breakpoints or left unused were plotted according to their median SHAPE reactivity value.

Figure S1

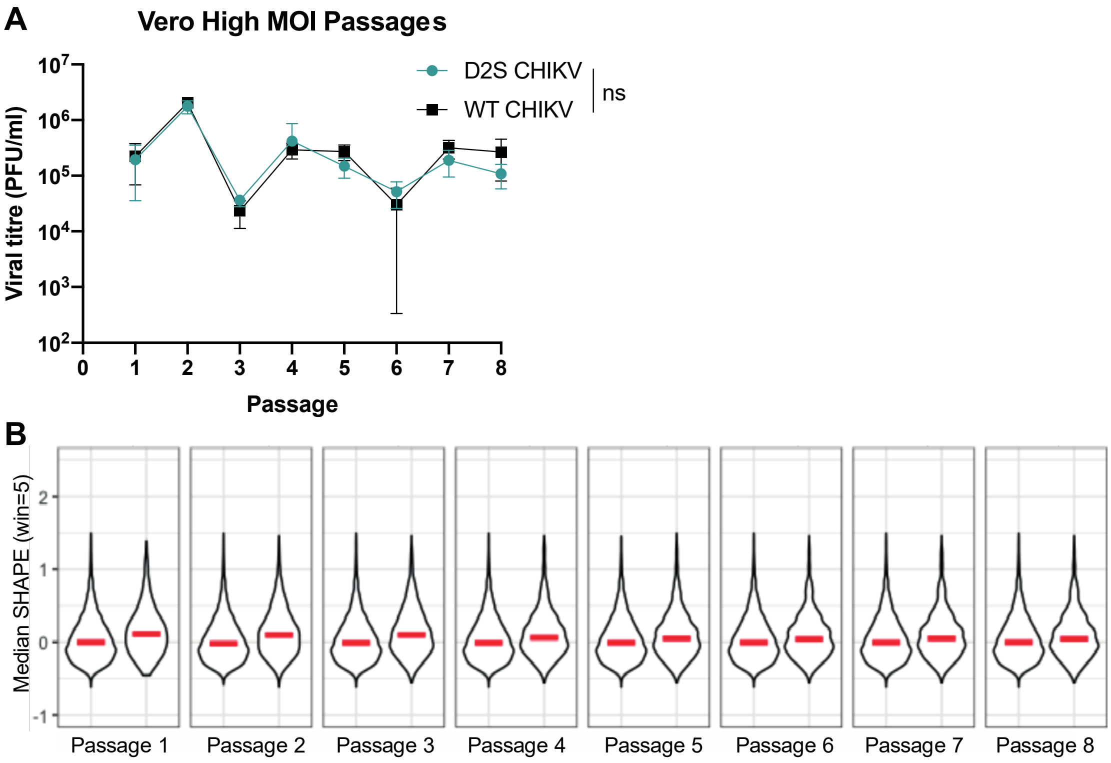

Supplementary Figure S2: Heatmaps of deleted nucleotide during WT or D2S passages

Heatmaps of deleted nucleotide during WT (A) or D2S (B) passages in Vero (6 replicates pooled together for each passage). Relative frequency of deleted nucleotides per million is depicted in shades of brown.

Figure S2

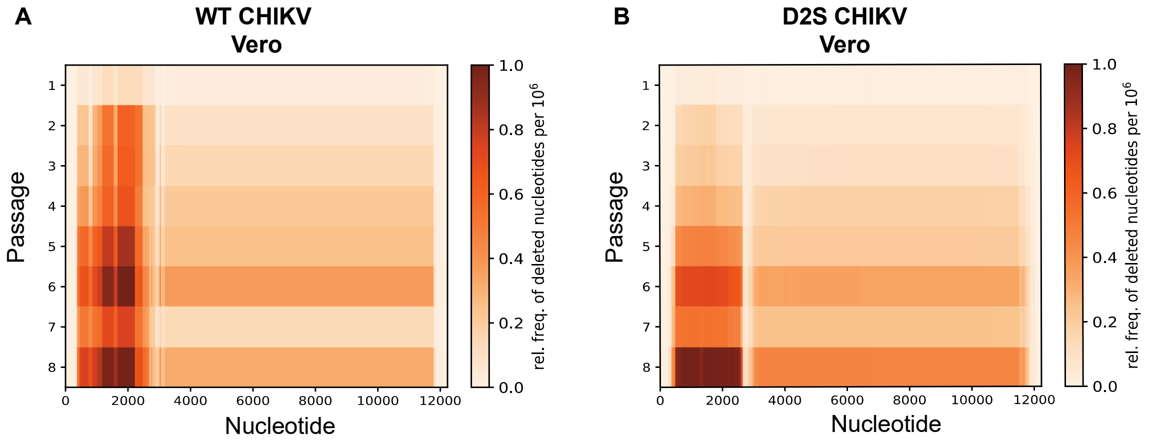

Table S1: List of mutation introduced in CHIKV Carib to generate D2S mutant.

Region 1 (cluster A and B start)	Region 2 (cluster A stop, cluster C start)	Region 3 (Cluster C stop)
A382T	T2818A	T3808A
G385C	A2821C	T3814C
T398C	A2827C	T3817A
A412T	A2839C	A3826G
A439G	T2848A	G3829C
T460G	C2851T	T3841C
C511A	G2857A	A3844T
T523A	A2872T	C3847A
G529A	T2875C	C3848T
A622G	G2884A	C3865T
T634C	A2887C	G3880C
G658A	C2890T	C3892A
T667A	C2896A	A3895C
C670T	A2905G	C3901A
A676G	T2911C	T3902C
C679T	C2923A	C3922G
T689C	A2926G	A3923T
A697T	A2932G	G3924C
G730A	G2941A	T3925C

T2947A	C3932T
C2956A	A3934G
C2962T	C3946T
A2965T	
G3046A	
T3049G	
A3082C	
C3085T	
C3092G	
G3097A	
G3121T	
A3124G	
T3142C	
G3145A	
C3170A	
A3184G	

Table S2: List of primers used to clone D2S mutant

	Insert primers	Vector primers
**5' CV1 start**	CTAATTATGCGAGAAAGCTCGCATC	GATGCGAGCTTTCTCGCATAATTAGC
**3' CV1 start**	GGTGCATGTACAGCATAGACGTC	GACGTCTATGCTGTACATGCACC
**5' CV2 start**	GTTCGACACAACCCCATTCATGT	GGGGTTGTGTCGAACCCTACC
**3' CV2 start**	GCCTCGTCTACCTTCCGTCAG	GAAGGTAGACGAGGCAAATTGTCT
**5' CV2 stop**	CGAGGTCATGACAGCTGCA	GCTGTCATGACCTCGTGTCC
**3' CV2 stop**	TCTTTATCCACGGGTCTCCGGAG	ACCCGTGGATAAAGACGCTGCA
**5' CV1 stop**	GTGCTCCACCTCCCACTCC	GTGCTCCACCTCCCACTCC
**3' CV1 stop**	ATGCTTTGTCTTCTTTGAAAGCTTGAATT	CAAAGAAGACAAAGCATACTCACCCG
**5' CV3/4 stop**	ACTGCAAATGCTAGGTGGTG	CCTAGCATTTGCAGTTTCATTGCG
**3' CV3/4 stop**	AACTGCTAAATAGGAAGAACATCTCAGT	CTTCCTATTTAGCAGTTTTGACAATGGC
